# Multiple Stressors in Aquatic Ecosystems: Sublethal Effects of Temperature, Dissolved Organic Matter, Light and a Neonicotinoid Insecticide on Gammarids

**DOI:** 10.1007/s00128-020-02926-6

**Published:** 2020-07-08

**Authors:** Mirco Bundschuh, Jochen P. Zubrod, Lara L. Petschick, Ralf Schulz

**Affiliations:** 1 iES Landau, Institute for Environmental Sciences, University of Koblenz-Landau, Fortstraße 7, 76829 Landau, Germany; 2 Eußerthal Ecosystem Research Station, University of Koblenz-Landau, Birkenthalstraße 13, 76857 Eußerthal, Germany; 3 Department of Aquatic Sciences and Assessment, Swedish University of Agricultural Sciences, Lennart Hjelms väg 9, 75007 Uppsala, Sweden

**Keywords:** Amphipod, Feeding rate, Multiple stress, Leaf litter decomposition

## Abstract

Whether and to which extent the effects of chemicals in the environment interact with other factors remains a scientific challenge. Here we assess the combined effects of temperature (16 vs. 20°C), light conditions (darkness vs. 400 lx), dissolved organic matter (DOM; 0 vs. 6 mg/L) and the model insecticide thiacloprid (0 vs. 3 µg/L) in a full-factorial experiment on molting and leaf consumption of *Gammarus fossarum*. Thiacloprid was the only factor significantly affecting gammarids’ molting. While DOM had low effects on leaf consumption, temperature, light and thiacloprid significantly affected this response variable. The various interactions among these factors were not significant suggesting additivity. Only the interaction of the factors temperature and thiacloprid suggested a tendency for antagonism. As most stressors interacted additively, their joint effects may be predictable with available models. However, synergistic interactions are difficult to capture while being central for securing ecosystem integrity.

The impact of chemical stressors in aquatic ecosystems is increasingly assessed against the background of multiple stress (e.g. Bracewell et al. 2019). Thereby research goes beyond the traditional focus on single substances and their impact on different levels of ecological complexity, that is from genes to ecosystems or even meta-ecosystems. Research is available, amongst others, on the interactions of effects induced by chemical stressors in combination with nutrients (Fernandez et al. 2016; Nuttens et al. 2016), food scarcity (Pereira and Goncalves 2007), salinity (Szöcs et al. 2012), suspended soil particles (Magbanua et al. 2013a, b), UV irradiation (Pelletier et al. 2006), temperature changes (Janssens et al. 2014) or daily temperature fluctuations (Verheyen et al. 2019). Most of the named additional factors can be related to changes in land use as well as climate change.

The direction and magnitude of the joint effects of multiple stressors in aquatic ecosystems is not clear cut with additive, synergistic and antagonistic interactions being reported (Jackson et al. 2016). For instance, Janssens et al. (2014) documented stronger effects of chlorpyrifos on the damselfly *Ischnura elegans* at 30°C relative to 20°C. For the same species, Op de Beeck et al. (2018) documented a higher impact of the same stressor at 20°C relative to 24°C. Op de Beeck et al. explained their findings with a slower degradation of chlorpyriphos at 20°C relative to 24°C leading to stronger effects. This interpretation contradicts, however, Janssens et al. (2014) showing a higher effect at 30°C despite an anticipated much faster degradation. This discrepancy may, however, be explained by the heat stress overriding the potentially positive effect of a faster chlorpyriphos degradation at 30°C. These two studies provide an excellent example for the complexity of interactions that are likely to play a significant role when assessing for effects of chemical stressors in a multiple stressor environment.

Besides these anthropogenically induced changes in environmental variables, the presence of dissolved organic matter (DOM) can have a substantial impact in the bioavailability of chemical stressors and their subsequent effects (Kukkonen and Oikari 1991). Moreover, long-term monitoring data suggest that DOM levels in surface waters are increasing (brownification) in regions with reduced sulfur deposition and wetter climate (de Wit et al. 2016). At the same time, DOM is hardly amended during ecotoxicological assessments in general making its impact on chemical stressor induced effects a substantial knowledge gap – particularly in a multiple stressor context. Similarly, light can influence organisms, particularly those with a negative phototaxis (Franke 1977), which in turn can be modified by stressors such as parasites (Benesh et al. 2005).

Using molting and leaf consumption of a key species in the ecosystem function of leaf litter decomposition, that is *Gammarus fossarum* (Dangles et al. 2004), the present study assessed the individual and combined effects of four factors in a full-factorial test design. Among those factors, DOM (0 vs. 6 mg/L) and light intensity (darkness vs. 400 lx) covered environmental variables and increasing temperature (16 vs 20°C) as well as the neonicotinoid insecticide thiacloprid (0 vs. 3 µg/L) represented factors modified in aquatic ecosystems as a consequence of global climate change and agricultural land use. The tested thiacloprid concentrations are considered field relevant as similar levels have been found in agricultural streams (Morrissey et al. 2015; Süss et al. 2006). It was hypothesised that light would reduce gammarids’ leaf consumption as they would hide under the leaf material (Bundschuh et al. 2011). Similarly, thiacloprid reduces this variable (Feckler et al. 2012) with DOM having, driven by the rather low hydrophobicity of thiacloprid, a slight mitigating impact (Kukkonen and Oikari 1991). Based on the review by Holmstrup et al. (2010), who suggested mainly synergistic effects between higher temperatures and chemical stressors, and thiacloprids stability in aquatic systems (Lewis et al. 2016), it was finally assumed that the impact of thiacloprid would be elevated at higher temperatures.

## Materials and Methods

The experiment followed a full-factorial design with four independent variables (temperature, light, DOM, toxicant) and two levels each. The factors temperature and light where realised in climate chambers. Temperature was set at 16 ± 1 or 20 ± 1°C to simulate a projected temperature increase under the A1B scenario (IPCC 2007). Light intensity was either approximately 400 lx – a moderate light intensity (Hubert et al. 1998) – with a light:dark cycle of 8:16 h or complete darkness. Seaweed extract (Marinure^®^, Glenside, Scottland) served as DOM source at a concentration of 6 mg/L, which is in the lower range of environmentally relevant levels (Evans et al. 2017). Seaweed extract was selected because of its relatively balanced composition in terms of chromophoric dissolved organic carbon, which is also representative for DOM released from waste water treatment plants. Moreover, it is recommended for chronic standard tests with Daphnids (OECD 2008). Thiacloprid was applied as commercially available formulation (Calypso^®^ 480 SC; 480 g thiacloprid/L; Bayer CropScience, Leverkusen, Germany; containing 1,2-Benzisothiazol-3(2H)-on, 5-Chlor-2-methyl-2H-isothiazol-3-on and 2-methyl-2H-isothiazol-3-on below 0.05% of the product each), which rendered the use of further solvents unnecessary. The formulation was serially diluted in amphipod medium (SAM-5S; Borgmann 1996) to receive the respective nominal concentration of 3 µg/L, a concentration that should cause a reduction in gammarids’ leaf consumption of about 50% over 7 days (Feckler et al. 2012). To verify exposure at these nominal concentrations, at the start of the experiments triplicate 10-mL samples were taken from the thiacloprid treatments. Samples were stored at − 20°C until analysis via an ultra-high-performance liquid chromatography system (Englert et al. 2012). The analyses revealed adequate thiacloprid dosing (mean ± standard deviation: 2.5 ± 0.3 µg/L) since nominal and measured initial concentrations deviated by < 20%.

Leaf discs were prepared as described in Zubrod et al. (2010). Briefly, shortly before leaf fall in October 2015, black alder (*Alnus glutinosa* (L.) Gaertn.) leaves from trees near Landau, Germany (49° 11′ N; 8° 05′ E) were collected and stored at − 20°C until use. Discs of 2 cm diameter were cut from the leaves with a cork borer, while excluding the main vein. Leaf discs were subjected to microbial colonization (i.e., conditioning) for 10 days in a nutrient medium (Dang et al. 2005) using leaf material previously exposed in a near-natural stream (Rodenbach, Germany, 49° 33′ N, 8° 02′ E) as inoculum. After conditioning, leaf discs were dried to a constant weight (~ 24 h at 60°C) and weighed to the nearest 0.01 mg. Approximately 48 h prior to the start of the experiment, leaf discs were re-soaked in sterile test medium (147 mg/L CaCl2 × 2H2O, 85.5 mg/L NaHCO3, 61.5 mg/L MgSO4 × 7H20, 3.8 mg/L KCl, and 1.03/L mg NaBr, Borgmann 1996) to prevent floating during the experiments.

The test organisms, *G. fossarum*, were kick-sampled in the near-natural, forested stream Hainbach near Landau, Germany (49° 14′ N; 8° 03′ E; cryptic lineage B; Feckler et al. 2014) 7 days prior to the experiment. Gammarids were immediately divided into different size classes (Franke 1977) and only adults with a diameter between 1.6 and 2.0 mm being visually free of acanthocephalan parasites were used in order to reduce variability in their response (Pascoe et al. 1995). At the start of the experiment gammarid populations were in a reproductive rest. Consequently, separation between sexes was not possible and the experiment was run with both males and females. Throughout the acclimation phase, animals were kept in aerated medium in a climate-controlled chamber at 16 ± 1 or 20 ± 1°C (depending on the temperature treatment) in total darkness, while they were fed ad libitum with pre-conditioned black alder leaves and were gradually adapted to SAM-5S.

The assessment of gammarids’ leaf consumption followed Zubrod et al (2010). One *G. fossarum* was placed together with two preconditioned leaf discs in a 250-mL-glass beaker filled with 200 mL of SAM-5S. Each of the 16 treatments of the four-factorial test design were replicated 20 times. All beakers were aerated during the whole study duration. In addition to the replicates established to quantify gammarids’ leaf consumption, four additional replicates were set up per treatment without gammarids to account for the microbial and physical leaf mass loss. During the study, molting was monitored daily. Amphipods, the remaining leaf discs and any leaf tissue shredded off were removed after seven days of exposure, dried and weighed as described above.

Leaf consumption was expressed in consumed leaf mass and according to Maltby et al. (2000). As the leaf consumption data did not meet the requirements of parametric testing, data were rank transformed and subsequently analysed by four-way analysis of variance (ANOVA). The proportion of moulting gammarids were analysed using a binomial generalized linear model (GLM). Both models were fitted excluding four-way interactions as these are hardly interpretable and such high-level interactions were not hypothesized. ANOVA and GLM were simplified stepwise using F-tests and chi-square tests to justify simplification steps, respectively. The term “significant(ly)” is exclusively used in reference to statistical significance (*p* < 0.05) throughout the present study. For all statistics and figures, R version 3.3.2 for Mac was used.

## Results and Discussion

**Fig. 1:**
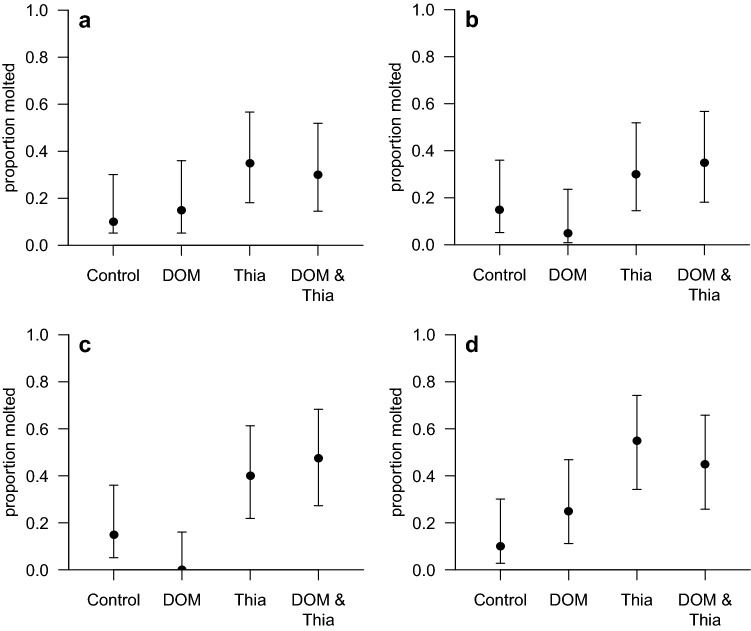
Proportion (with 95% confidence interval) of molted gammarids exposed to DOM, thiacloprid (Thia) and their combination at 16°C (**a**, **b**) and 20°C (**c**, **d**) in presence (**a**, **c**) and absence (**b**, **d**) of light

The only factor significantly affecting gammarids’ molting was the presence of thiacloprid during the experiment increasing the proportion of molted animals on average by more than three-fold compared to the thiacloprid-free treatments (Fig. 1; Table 1). This observation, may be related to detoxification, namely reducing the concentration of thiacloprid by molting – a mechanism observed for other stressors such as metals (Raessler et al. 2005). The present study, however, contrasts earlier studies reporting a reduction in molting rate, for instance, in cladoceran *Daphnia magna* (Qi et al. 2013) or the mayfly *Deleatidium* spp. (Macaulay et al. 2019) at higher concentrations of other neonicotinoids, namely guadipyr, imidacloprid, thiamethoxam and clothianidin. These contrasting effects point towards species- or neonicotinoid-specific effects in molting requiring further research to uncover the underlying mechanisms.

**Table 1:** Minimal adequate model resulting from simplifying four-way GLM performed on moulting data

	Estimate	Std. error	z-value	*p*-value
Intercept	− 2.0043	0.2444	− 8.202	< 0.001
Thiacloprid	1.5831	0.2933	5.398	< 0.001

**Fig. 2:**
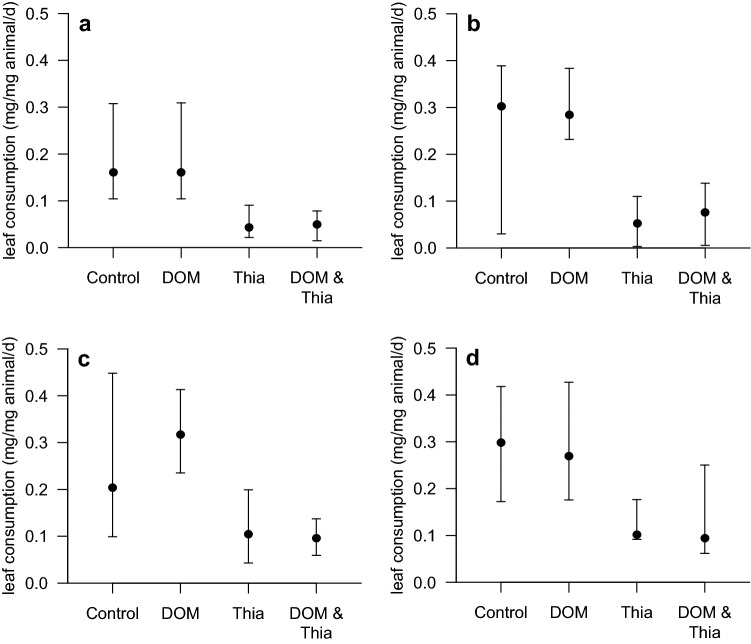
Median (with 95% confidence interval) leaf consumption (mg/mg dry weight of gammarids/d) of gammarids exposed to DOM, thiacloprid (Thia) and their combination at 16°C (**a**, **b**) and 20°C (**c**, **d**) in presence (**a**, **c**) and absence (**b**, **d**) of light

The median leaf consumption of *G. fossarum* was, in absence of thiacloprid and DOM, reduced by roughly 40% under light exposure relative to those tested in darkness (Fig. 2a–d; Table 2). Moreover, the effect size matched roughly with the light:dark rhythm of 8:16 h. This finding, thus, confirms one of the hypotheses, namely that light reduces gammarids leaf consumption likely driven by their negative phototaxis and tendency to hide under leaf litter instead of consuming it (see also Bundschuh et al. 2011).

**Table 2:** Minimal adequate model resulting from simplifying four-way ANOVA performed on rank-transformed leaf consumption data

	Df	Sum of squares	Mean squares	F-value	*p*-value
Thiacloprid	1	582,851	582,851	107.32	< 0.001
Temperature	1	99,771	99,771	18.371	< 0.001
Light	1	29,276	29,276	5.391	0.021
Residuals	300	1,629,282	5431		

The presence of DOM was, relative to its absence, inducing no changes in leaf consumption under most light and temperature regimes applied (Table 2, Fig. 2a–d). Only at 20°C in presence of light, leaf consumption was substantially elevated, while overall DOM was a non-significant factor for explaining variability in gammarids’ feeding. This observation contradicts recent studies with *Daphnia* reporting in absence of UV light increasing DNA strand breaks with increasing DOM concentrations (Wolf et al. 2017). The concentrations causing these negative effects were, however, several fold above the levels applied in the present study. Moreover, DNA strand breaks are suborganismic responses that may be balanced on the level of whole organisms leading to insubstantial effects on behavioural responses as shown here.

Temperature significantly affected leaf consumption (Table 2), with the higher temperature (20°C) leading to higher leaf consumption relative to 16°C (Fig. 2a–d). A higher activity of organisms at higher temperatures (up to a individuum specific threshold) was also observed in a range of other studies (e.g. Cuco et al. 2018; Seeland et al. 2013) highlighting that the selected temperature range in the present study was still acceptable for our tested population. This difference within the same treatment between temperatures can be as high as 2.5-fold (thiacloprid in light) or close to zero (in darkness for both control and DOM). The most consistent effect of temperature was observed in presence of thiacloprid with effect sizes often around a factor of two (but not in darkness in presence of DOM and thiacloprid). The only interaction term near to significance (*p* = 0.058; F-test justified omission from final model; Table 2) was the interaction between temperature and thiacloprid pointing towards a lower relative reduction in gammarid feeding at 20°C relative to 16°C. Thereby, evidence against one hypothesis, namely that elevated temperatures increase the impact of pesticides, was found. This positive effect may be explained by the fact that the used temperature of 20°C is still within the range that can be accepted by *G. fossarum* (Peeters and Gardeniers 1998). Consequently, gammarids’ defence mechanisms may have been more efficient with a higher enzyme activity at 20°C, while this temperature did not have a direct negative impact on the test species.

Thiacloprid reduced leaf consumption by partly more than 80%. This massive effect of the neonicotinoid is supported by the four-way ANOVA highlighting this factor as significant (Table 2). The effect induced by 3 µg thiacloprid/L is in line with earlier studies reporting slightly lower but in general similar effects on gammarids (Englert et al. 2012; Feckler et al. 2012). The data additionally supports the related initial hypothesis. Any interaction with DOM and light (i.e., independent whether two or threefold interaction) was not significant suggesting additive effects of these factors with thiacloprid on gammarids’ leaf consumption (Table 2). The low impact of DOM on thiacloprid-induced effects is, as hypothesised, likely triggered by its low affinity to organic carbon and lipophilicity (Lewis et al. 2016). However, DOM is also considered as additional energy source for aquatic invertebrates such as daphnids (Bergman Filho et al. 2011) but also black fly larvae (Ciborowski et al. 1997) potentially leading to a higher tolerance to chemical stress (Seitz et al. 2015). The data of the present study suggest DOM, but also light, to play a minor role for the bioavailability of the assessed insecticide as well as for the tolerance of the test species.

All in all, the present study highlights that none of the factor combinations lead to significant interactions. Although this might be a matter of statistical power, the data suggest additive effects on the assessed variables. Consequently, the joint effects of the factors assessed in the present study should be predictable with available models. At the same time, synergistic interactions between natural factors but also those of anthropogenic origin have been reported (Holmstrup et al. 2010; Jackson et al. 2016) complicating reliable predictions. This fact warrants further research, which should consider the mechanistic framework proposed by Schäfer and Piggott (2018).
